# Using mechanistic physiologically-based pharmacokinetic models to assess prenatal drug exposure: Thalidomide *versus* efavirenz as case studies

**DOI:** 10.1016/j.ejps.2019.105068

**Published:** 2019-12-01

**Authors:** Shakir Adeyinka Atoyebi, Rajith K.R. Rajoli, Ebunoluwa Adejuyigbe, Andrew Owen, Oluseye Bolaji, Marco Siccardi, Adeniyi Olagunju

**Affiliations:** a Department of Pharmaceutical Chemistry, Obafemi Awolowo University, Ile-Ife, Nigeria; b Department of Molecular and Clinical Pharmacology, University of Liverpool, United Kingdom; c Department of Paediatrics and Child Health, Obafemi Awolowo University, Ile-Ife, Nigeria

**Keywords:** PBPK model, Efavirenz, Thalidomide, Pregnancy, Fetus

## Abstract

Maternofoetal physiologically-based pharmacokinetic models integrating multi-compartmental maternal and foetal units were developed using Simbiology® to estimate prenatal drug exposure. Processes governing drug disposition were described using differential equations with key system and drug-specific parameters. Transplacental drug transfer was modelled as bidirectional passive diffusion and benchmarked against those for thalidomide as a control. Model-predictions for pharmacokinetic parameters during pregnancy were within acceptable ranges for qualification (two-fold difference of clinically-observed values). Predicted foetal exposure to thalidomide was higher than efavirenz, with median (range) foetal-to-maternal plasma ratios of 4.55 (3.06–9.57) for 400 mg thalidomide *versus* 0.89 (0.73–1.05) for 400 mg efavirenz at third trimester. Model-predictions indicated foetal exposure consistently above 300% of maternal plasma concentration for thalidomide throughout pregnancy, while exposure to efavirenz increased from under 20% at second trimester to above 100% at third trimester. Further qualification of this approach as a tool in evaluating drug exposure and safety during pregnancy is warranted.

## Graphical abstract

**Figure:**
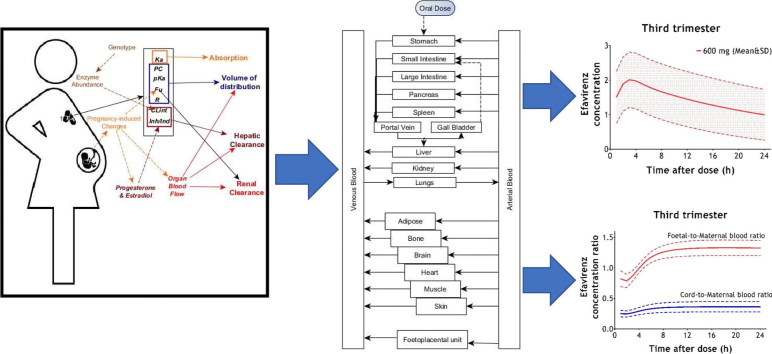

## 1 Introduction

Thalidomide was first marketed for its sedative effects which were non-addictive unlike the barbiturates. Because it was generally regarded as safe with some packs labelled as “free from untoward side-effects” (Vargesson, 2009), it was widely used to treat morning sickness during pregnancy due to its antiemetic properties. However, global reports of severe birth defects affecting the limbs (phocomelia), ear, eyes, heart, kidneys, genitals and CNS of babies whose mothers had ingested thalidomide during pregnancy caused its withdrawal from the market in 1962 (Vargesson, 2009). This also led to increased monitoring and regulation of all new drug applications. Though thalidomide is currently licensed only for the treatment of multiple myeloma and erythema nodosum leprosum, it has also been used as an investigational drug for cancer and certain HIV- associated symptoms under a very strict monitoring system; inadvertent exposures during pregnancy have been reported in Brazil (Vargesson, 2015).

Genuine concerns exist about the thin line, and sometimes overlap, between the potential benefits of drug use to the mother and risks of toxicity to the developing foetus during pregnancy. Stricter measures were introduced by regulatory bodies and fear of potential legal liability contributed to the exclusion of pregnant women from clinical trials, resulting in a scarcity of data on the efficacy and safety of most drugs in pregnant women (Blehar et al., 2013; Vargesson, 2015). Prescribers therefore often need to treat pregnant women with drug doses established in non-pregnant adults, which could be misleading due to the effects of pregnancy on the disposition of many drugs (Gaohua et al., 2012). Physiological changes during pregnancy such as variation in hepatic drug metabolising enzymes and increased glomerular filtration rates have been shown to influence the disposition of efavirenz (Olagunju et al., 2015a) and cefazolin (Dallmann et al., 2017) respectively. However, drug use during pregnancy is often unavoidable for both maternal and foetal health. For instance, HIV positive women continue antiretroviral drugs during pregnancy to maintain virological suppression and for the prevention of mother-to-child transmission (PMTCT) of HIV (WHO, 2016). No significant safety concerns have arisen for antiretroviral drugs currently used during pregnancy. For instance, despite early concerns about possible teratogenicity (WHO, 2009), the non-nucleoside reverse transcriptase inhibitor, efavirenz has been shown to be safe throughout pregnancy (Ford et al., 2014).

A major gap currently exists in our understanding of the link between the extent of foetal exposure to maternal drugs and reported toxicity or safety. Using thalidomide and efavirenz as case studies, the current work used physiologically-based pharmacokinetic modelling to estimate the extent of foetal exposure to maternal drugs during different stages of pregnancy.

## 2 Materials & methods

### 2.1 Model structure and parameterisation

The adult human and maternofoetal physiologically-based pharmacokinetic (PBPK/mf-PBPK) models employed for this study were built using Simbiology® (v. 5.7, MATLAB® 2017b, Mathworks Inc., Natick, Massachusetts, USA). The female adult PBPK model was created by modifying a previously validated breastfeeding human whole-body PBPK model for orally-administered efavirenz (Olagunju, 2015). The individual organ weights in the adult model were predicted anthropometrically as previously reported (Bosgra et al., 2012), while the anthropometric characteristics such as age and height were based on data collected from a cohort of HIV-infected breastfeeding women(Bosgra et al., 2012; Olagunju et al., 2015b).

The mf-PBPK model is composed of the female adult model integrated with a foetal PBPK sub-model which was built based on a newly reported maternal-foetal PBPK model template (Zhang et al., 2017). Gestational-age dependent anatomical and physiological parameters were incorporated into the model for both the maternal and foetal compartments (Abduljalil et al., 2012; Zhang et al., 2017). The pattern of the foetal blood circulation and the fractional blood flow of the cardiac output to maternal organs were based on data reported in Annals of the International Commission on Radiological Protection (ICRP) (ICRP, 2003). The drug-specific parameters incorporated into the model are presented in Table 1.

**Table 1:** Drug-specific parameters for thalidomide and efavirenz.

Drug properties	Description	Thalidomide	Efavirenz (Rajoli et al., 2015)
MW (g)	Molecular weight	258 (Olagunju et al., 2015b)	316
LogP	Octanol-water partition coefficient	0.528 (Nishiyama et al., 2015)	4.60
pKa	Acid dissociation constant	11.59 (Olagunju et al., 2015b)	10.2
R	Blood:plasma drug ratio	0.878 (Nishiyama et al., 2015)	0.74
PSA	Polar surface area	83.55 (Olagunju et al., 2015b)	38.33
HBD	Hydrogen bond donor	1 (Olagunju et al., 2015b)	1
f_U_	Fraction unbound	0.635 (Nishiyama et al., 2015)	0.015
V_d_ (L/kg)	Volume of distribution	–	3.6
P_app_ (10^−6^ cm/s)	Drug permeability in Caco-2 monolayer	–	2.5
K (10 cm^2^/s)	Diffusion constant	1.10^b^	0.25^a^
CL_int_ (μL/min/pmol)	Intrinsic hepatic clearance		
rCYP1A2 CL_int_		–	0.008
rCYP2A6 CL_int_		–	0.05
rCYP2B6 CL_int_		–	0.55
rCYP2C19 CL_int_		0.00029^c^	–
rCYP3A4 CL_int_		–	0.007
rCYP3A5 CL_int_		–	0.03
Ind_CYP_ (μM)	Hepatic CYP induction		
CYP2B6 Ind_max_		–	5.76
CYP2B6 Ind_50_		–	0.82
CYP3A4 Ind_max_		–	6.45
CYP3A4 Ind_50_		–	3.93
CL_hyd_ (L/h)	Clearance by hydrolysis	14.48^d^	–

a Model-fitted through sensitivity analysis shown on Table S1.

b Extrapolated from efavirenz using P_eff_ as shown in Eq. (11).

c Calculated using previously reported data (Lu et al., 2004).

d Calculated using previously reported data (Lepper et al., 2006; Nishiyama et al., 2015).

The foetal sub-model comprises of compartments representing the placenta, the amniotic fluid, the foetal kidney, foetal liver and foetal brain while other foetal organs were lumped into a single compartment as previously described (Zhang et al., 2017). The equations used to define the blood flow to the foetal organs represented in the model were as described by Zhang et al. (Zhang et al., 2017). The blood flow through the portal sinus was described as the difference between the blood flow through the umbilical vein and the ductus venosus, while the blood flow through the ductus arteriosus and foramen ovale were obtained by digitising data reported in literature (Sutton et al., 1994). The variation of the placental thickness during gestation was obtained from literature (Karthikeyan et al., 2012). The graph-plots showing the variation of the parameters with gestational age were digitised in the absence of raw data using Plotdigitizer® version 2.6.6 (Free Software Foundation, Boston, MA, USA). The generated raw input points were plotted and analysed on Microsoft Excel® (Microsoft Inc., Redmond, Washington, US) to obtain the equations of best-fit which were subsequently inputted into the model.

### 2.2 Absorption

The drug ADME were described similarly to the previous breastfeeding model (Olagunju, 2015). The drug absorption was described using a compartmental absorption and transit model incorporating both gastric emptying and small intestinal transit flow. The effective permeability (P_eff_) used for the estimation of the absorption rate constant (K_a_) was derived from Caco-2 permeability or polar surface area and number of hydrogen bond donors as previously described (Siccardi et al., 2013; Yu and Amidon, 1999).

(1) $$P_{\mathrm{eff}}=10^{\left(0.6836\times \left(\log\;\mathrm{Caco}-2\right)–0.5579\right)}$$

(2) $$P_{\mathrm{eff}}=10^{\left(-2.546–0.011\;\left(\mathrm{PSA}\right)–0.278\;\left(\mathrm{HBD}\right)\right)}$$

(3) $$K_{a}=\frac{2\;P_{\mathrm{eff}}}{R}$$

where R is the radius of the small intestine.

### 2.3 Distribution

The systemic drug circulation was defined as perfusion-rate limited (*i.e.* as a function of the blood flow-rate to the tissues/organs). The equations describing the systemic drug circulation and the volume of distribution (V_SS_) have been previously published (Peter, 2008; Poulin and Theil, 2002).

(4) $$\mathrm{Pt}:p,\mathrm{nonadipose}=\frac{\left[\mathrm{Po}:w\times \left(\mathrm{Vnlt}+0.3\times \mathrm{Vpht}\right)\right]+\left[1\times \left(\mathrm{Vwt}+0.7\times \mathrm{Vpht}\right)\right]}{\left[\mathrm{Po}:w\times \left(\mathrm{Vnlp}+0.3\times \mathrm{Vphp}\right)\right]+\left[1\times \left(\mathrm{Vwp}+0.7\times \mathrm{Vphp}\right)\right]}\times \frac{\mathrm{fu},p}{\mathrm{fu},t}$$

(5) $$\mathrm{Pt}:p,\mathrm{adipose}=\frac{\left[\mathrm{Dvo}:w\times \left(\mathrm{Vnlt}+0.3\times \mathrm{Vpht}\right)\right]+\left[1\times \left(\mathrm{Vwt}+0.7\times \mathrm{Vpht}\right)\right]}{\left[\mathrm{Dvo}:w\times \left(\mathrm{Vnlp}+0.3\times \mathrm{Vphp}\right)\right]+\left[1\times \left(\mathrm{Vwp}+0.7\times \mathrm{Vphp}\right)\right]}\times \frac{\mathrm{fu},p}{1}$$

where Pt:p,adipose is adipose tissue:plasma partition coefficient; Pt:p, nonadipose is nonadipose tissue:plasma partition coefficient; Po:w is n-octanol:buffer partition coefficient of the non-ionised species at pH 7.4; Dvo:w is olive oil:buffer partition coefficient of the ionised and non-ionised species at pH 7.4; V is fractional tissue volume content of neutral lipids (nl), phospholipids (ph) and water(w); t is tissue and p is plasma.

(6) $$V_{\mathrm{ss}}=\left(\mathrm{\Sigma Vt}*\mathrm{Pt}:p\right)+\left(\mathrm{Ve}*E:P\right)+\mathrm{Vp}$$

where V_SS_ is Volume of distribution at steady state; V is fractional body volume of erythrocyte (e), plasma (p) and tissue (t); E:P is erythrocyte:plasma ratio.

The effect of pregnancy on the fraction of the unbound drug in the maternal and foetal compartment was incorporated into the model and calculated with respect to plasma concentrations of plasma proteins which vary with gestational age as previously described (Dallmann et al., 2017).

(7) $$F_{u,p}=\frac{1}{1+\left(K_{p,\mathrm{pp}}\times \left[P_{\mathrm{pp}}\right]\;\right)}$$

(8) $$K_{p,\mathrm{pp}}=\frac{\left(1-f_{u}\;\right)}{\left(69.7\times f_{u}\right)}$$

where f_u_ is the fraction of the unbound drug in non-pregnant adults; [P_pp_] is the concentration of plasma proteins in pregnancy; F_u,p_ is the fraction of the unbound drug during pregnancy and K_p,pp_ is the constant of association of the drug to plasma proteins.

### 2.4 Metabolism and elimination

The main methods of elimination of thalidomide and efavirenz which are through plasma hydrolysis and hepatic clearance, mediated by CYP450 enzymes, respectively, were both fitted into the model. The reported half-life (t_1/2_) of *in vitro* hydrolysis (Lepper et al., 2006) and volume of distribution (V_d_) of thalidomide in humanised mice (Nishiyama et al., 2015) were used to estimate the systemic clearance of thalidomide by plasma hydrolysis as described below. The clearance of thalidomide by plasma hydrolysis in the foetal model was scaled from the maternal model using maternal and foetal plasma volumes.

(9) $$\mathrm{CL}=\frac{V_{d}\times 0.693}{t_{1/2}}$$

The intestinal metabolism of efavirenz and the hepatic clearance of efavirenz mediated by CYP450 enzymes were scaled from *in vitro* to *in vivo* in the maternal model. The same technique was applied to modulate the metabolism of both drugs in foetal liver within the foetal compartment. The intrinsic drug clearance of each enzyme was assumed to be similar between the maternal and foetal enzymes. The metabolism of thalidomide by CYP2C19, though negligible, was also incorporated (Lu et al., 2004). The equations describing the intestinal and hepatic clearance of drugs by CYP450 enzymes have been previously published (Rajoli et al., 2015; Siccardi et al., 2013). The enzyme abundance of CYP2B6 and CYP3A7 in foetal liver during gestation were derived from published data (Croom et al., 2009; Hines, 2007). CYP2B6 and CYP3A4 induction by efavirenz plasma concentration, as previously described (Siccardi et al., 2013), were incorporated into the model. Similarly, CYP2B6 induction by varying plasma levels of estradiol during pregnancy was incorporated into the model as described by Dickmann and Isoherranen (2012).

### 2.5 Modelling foetal exposure to maternal drugs

The transplacental drug transfer was modelled as bidirectional passive diffusion based on an adaptation of Fick's Law of diffusion (Griffiths and Campbell, 2015).

(10) $$Q_{\mathrm{pl},\mathrm{drug}}=\frac{K\times SA_{\mathrm{pv}}\times f_{u}\times \left(C_{1}-C_{2}\right)}{\mathrm{PT}}\;$$

where K is the diffusion constant; SA_pv_ is the placental villous surface area; f_u_ is the fraction of the unbound drug; (C_1_ – C_2_) is the concentration gradient across the placenta; and PT is the placental thickness. The fraction of the unbound drug dependent on the plasma proteins levels in the foetal compartment was used to calculate the foetal-to-maternal transplacental drug transfer and *vice versa*.

The diffusion constant for efavirenz was fitted into the model using sum of residuals through sensitivity analysis (Table S1). The diffusion constant of thalidomide was determined by extrapolation from efavirenz as shown in the equation below:

(11) $$K_{\mathrm{THAL}}=\frac{P_{\mathrm{eff},\mathrm{THAL}}\times K_{\mathrm{EFV}}}{P_{\mathrm{eff},\mathrm{EFV}}}$$

where P_eff_ is the effective permeability and K is the diffusion constant.

The equations defining the blood flow to the foetal organs represented in the model were generally obtained from data previously reported (Zhang et al., 2017). For instance, the blood flow through the foetal portal vein (Qpv) was described using gestational age (GA) with this equation:

(12) $$\mathrm{Qpv}\;\left(L/h\right)=0.714+0.0489\;\mathrm{GA}+0.0008\;{\mathrm{GA}}^{2}$$

The digitised data for foramen ovale and ductus arteriosus blood flow obtained from Sutton et al. (Sutton et al., 1994) were used to derive equations dependent on gestational age. For instance, the ductus arteriosus blood flow (Qda) was described as:

(13) $$\mathrm{Qda}\;\left(L/h\right)=0.0056\;\mathrm{GA}+0.1441$$

The extent of foetal exposure was estimated using the ratios of time-averaged drug concentration in cord plasma to maternal plasma (cord-to-maternal plasma ratio) and foetal plasma to maternal plasma (foetal-to-maternal plasma ratio).

### 2.6 Model validation

The mean simulated values of the system parameters were compared with available reference values reported in literature. Data reported in literature were used for the validation of simulated values of maternal organ weights (Molina and DiMaio, 2015). The foetal organ and other tissue weights were validated using data from Abduljalil et al. (2012) and Archie et al. (2006). The blood flows were validated using data in Abduljalil et al. (2012) and the ICRP (2003). The predicted thalidomide and efavirenz pharmacokinetic parameters were validated against published clinical data (Cressey et al., 2012; Dickinson et al., 2016; FDA, 2001; Gandhi et al., 2013; Olagunju et al., 2015a). Relevant studies with similar scenarios such as doses and pregnancy status were searched through PubMed using the following keywords: “pharmacokinetics”, “thalidomide”, “efavirenz” and “pregnancy”. The predicted pharmacokinetic parameters at steady-state were computed from simulated plasma concentration-time data using non-compartmental analysis on Microsoft Excel® (Microsoft Inc., Redmond, Washington, US). An acceptance threshold of two-fold difference between simulated and clinically observed values were set for both system and pharmacokinetic parameters.

## 3 Results

### 3.1 Validation of system parameters for pregnancy PBPK model

The key anatomical and physiological parameters such as organ weights, regional blood flow and CYP enzyme abundances in both maternal and foetal models were within a two-fold difference (*i.e.* simulated-to-reported ratios were between 0.5 and 2.0) when compared with available data. For example, the simulated mean weight of maternal liver, kidney and brain were 1.43, 0.28 and 1.31 kg respectively while the reported reference values were 1.40, 0.275 and 1.30 kg (ICRP, 2003).

### 3.2 Validation of model-predicted pharmacokinetic parameters for efavirenz and thalidomide in non-pregnant and pregnant women

The model-predicted pharmacokinetic parameters for 400 mg and 600 mg efavirenz in non-pregnant adults were within two-fold difference of clinically-observed figures with the maximum predicted-to-observed ratio of 0.98 for 400 mg efavirenz C_max_, and 1.1 for 600 mg efavirenz C_24_ (Table 2). The model-predicted pharmacokinetic parameters for 200 mg and 400 mg thalidomide in non-pregnant adults were also within two-fold difference of observed clinical values with maximum predicted-to-observed ratio of 1.2 for 200 mg thalidomide and 1.5 for 400 mg thalidomide (Table 2).

**Table 2:** Predicted *versus* observed plasma pharmacokinetics of efavirenz and thalidomide in non-pregnant adults.

Parameters	Observed	Predicted	Predicted/observed ratio
Efavirenz^a^	(Dickinson et al., 2016) n = 605	n = 100	
400 mg			
C_12_ (mg/L)	2.10 (2.01–2.20)	1.86 (1.65–2.06)	0.89
C_24_ (mg/L)	1.40 (1.32–1.49)	1.30 (1.10–1.49)	0.93
C_max_ (mg/L)	2.52 (2.42–2.62)	2.47 (2.27–2.67)	0.98
AUC_0–24_ (mg.h/L)	49.2 (47.0–51.5)	42.6 (38.0–47.2)	0.87
600 mg			
C_12_ (mg/L)	2.85 (2.70–3.0)	2.93 (2.59–3.27)	1.0
C_24_ (mg/L)	1.82 (1.68–1.97)	2.07 (1.75–2.40)	1.1
C_max_ (mg/L)	3.66 (3.51–3.81)	3.86 (3.52–4.20)	1.1
AUC_0–24_ (mg.h/L)	67.2 (63.8–70.9)	67.3 (59.5–75.0)	1.0
Thalidomide^b^	Thalomid Label_FDA (2001)	n = 100	
200 mg			
C_max_ (mg/L)	1.76 (30)	2.15 (17.7)	1.2
AUC_0–24_ (mg.h/L)	18.9 (17)	16.1 (18.6)	0.85
400 mg			
C_max_ (mg/L)	2.82 (28)	4.33 (18.2)	1.5
AUC_0–24_ (mg.h/L)	36.4 (26)	32.4 (18.5)	0.89

a Mean (90% CI) at steady-state.

b Mean (%CV) after single dose.

The model-predicted pharmacokinetics for 400 mg efavirenz in pregnant adults were within two-fold difference of clinically-observed figures with the maximum predicted-to-observed ratio of 0.89 for 400 mg efavirenz AUC_0–24_ (Table 3). The model-predicted pharmacokinetics for 600 mg efavirenz in the general population during pregnancy were compared with two sets of clinically-observed data with different duration within gestation: throughout pregnancy and during the third trimester. Using the data representing throughout pregnancy, the model-predicted pharmacokinetic parameters for 600 mg efavirenz were within a two-fold difference of clinically-observed data with the maximum predicted-to-observed ratio of 1.4 for efavirenz C_min_ (Table 3). Using the clinical data observed in the third trimester, the model-predicted pharmacokinetic parameters for 600 mg efavirenz were within two-fold difference of observed clinical values with the maximum predicted-to-observed ratio of 1.3 for efavirenz CL/F (Table 3).

**Table 3:** Predicted *versus* observed pharmacokinetics of efavirenz at steady-state during pregnancy.

Pharmacokinetic parameter (units)	Observed values	Simulated values	Predicted/observed ratio
400 mg efavirenz^a^			
Third trimester	(Lamorde et al., 2018) n = 25	n = 100	
C_min_ (mg/L)	1.21 (0.878–1.65)	1.07 (0.915–1.23)	0.88
C_max_ (mg/L)	2.75 (2.25–3.36)	2.11 (1.94–2.28)	0.77
AUC_0–24_ (mg.h/L)	39.9 (30.8–51.7)	35.6 (31.7–39.4)	0.89
600 mg efavirenz^b^			
Throughout pregnancy	(Olagunju et al., 2015a) n = 25	n = 100	
C_min_ (mg/L)	1.00 (0.429–5.19)	1.44 (0.303–8.61)	1.4
C_max_ (mg/L)	3.49 (1.26–14.4)	2.97 (1.50–9.82)	0.85
CL/F (L/h)	14.1 (2.96–27.7)	12.1 (2.84–32.5)	0.86
AUC_0–24_ (mg.h/L)	42.6 (21.7–203)	49.5 (18.4–211)	1.2
Third trimester	(Cressey et al., 2012) n = 25	n = 100	
C_min_ (mg/L)	1.60 (0.23–8.13)	1.20 (0.237–12.1)	0.75
C_max_ (mg/L)	5.44 (1.90–12.2)	2.72 (1.46–13.4)	0.50
CL/F (L/h)	10.8 (2.7–44.4)	13.8 (2.05–36.0)	1.3
AUC_0–24_ (mg.h/L)	55.4 (13.5–220)	43.5 (16.9–292)	0.79
At delivery			
Umbilical vein	(Cressey et al., 2012) n = 23	n = 100	
Efavirenz concentration (mg/L)	1.05 (0.47–4.51)	0.745 (0.341–3.84)	0.71
C:M ratio	0.49 (0.37–0.74)	0.47 (0.42–0.58)	0.97
Foetal plasma	(Gandhi et al., 2013) n = 50	n = 100	
Efavirenz concentration (mg/L)	1.70 (0.050–7.88)	1.47 (0.654–7.92)	0.86

a Data presented as mean (95% Confidence Interval).

b Data presented as median (range).

The model-predicted indices of foetal exposure to 600 mg efavirenz in the umbilical vein were within two-fold difference of clinically-observed figures, with the maximum predicted-to-observed ratio of 0.97 for cord-to-maternal plasma concentration ratio. Conversely, the model-predicted foetal plasma concentration at delivery after the maternal administration of 600 mg efavirenz were within two-fold difference of clinically-observed figures but the lower limit was over-predicted (Table 3).

Predicted data on the pharmacokinetics of 200 mg thalidomide, 400 mg thalidomide and 400 mg efavirenz in the pregnant women at second and third trimesters are shown in Table 4.

**Table 4:** Predicted pharmacokinetics of 400 mg efavirenz and thalidomide in the maternal plasma during pregnancy.

Pharmacokinetic parameter	Second trimester n = 100	Third trimester n = 100
400 mg efavirenz		
C_min_ (mg/L)	1.35 (0.460–5.91)	0.845 (0.269–4.98)
C_max_ (mg/L)	2.54 (1.30–6.92)	1.93 (0.977–5.94)
CL/F (L/h)	13.6 (4.06–31.6)	19.1 (4.77–46.8)
AUC_0–24_ (mg.h/L)	44.0 (19.0–148)	31.4 (12.8–126)

200 mg thalidomide		
C_min_ (mg/L)	0.070 (0.019–0.287)	0.073 (0.019–0.287)
C_max_ (mg/L)	1.97 (1.45–3.20)	1.97 (1.55–3.20)
CL/F (L/h)	12.3 (8.02–17.6)	12.2 (8.02–17.6)
AUC_0–24_ (mg.h/L)	16.2 (11.3–24.9)	16.4 (11.3–24.9)

400 mg thalidomide		
C_min_ (mg/L)	0.168 (0.036–0.670)	0.145 (0.038–0.573)
C_max_ (mg/L)	3.91 (2.87–6.56)	3.95 (3.11–6.40)
CL/F (L/h)	12.0 (6.91–18.9)	12.2 (8.02–17.6)
AUC_0–24_ (mg.h/L)	33.3 (21.2–57.9)	32.9 (22.7–49.8)

Data presented as median (range).

**Fig. 1:**
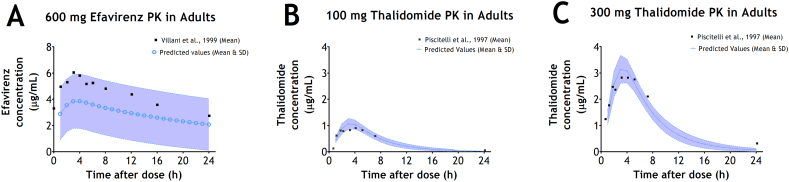
Predicted *vs* Observed plasma concentration-time profile of efavirenz and thalidomide in adults. A - Observed (mean) reported by Villani et al. (1999) and Predicted (mean & SD) plasma concentration-time profile of 600 mg efavirenz at steady-state in adults; and B - Observed (mean) reported by Piscitelli et al. (1997) and Predicted (mean & SD) plasma concentration-time profile of 100 mg thalidomide after single-dose in adults; and C - Observed (mean) reported by Piscitelli et al. (1997) and Predicted (mean & SD) plasma concentration-time profile of 300 mg thalidomide after single-dose in adults.

The overlay of the predicted and observed plasma concentration-time profiles of efavirenz and thalidomide are shown in Fig. 1. Fig. 1A shows the comparison between the mean plasma concentration-time profile of 600 mg efavirenz at steady state in adults as reported by Villani et al. (1999) and the corresponding predicted plasma concentration-time profile of 600 mg efavirenz in adults. Fig. 1B and C shows the comparison between the mean plasma concentration-time profiles of single doses of 100 mg and 300 mg thalidomide in adults as reported by Piscitelli et al. (1997) and the corresponding predicted plasma concentration-time profiles of 100 mg and 300 mg thalidomide respectively.

**Fig. 2:**
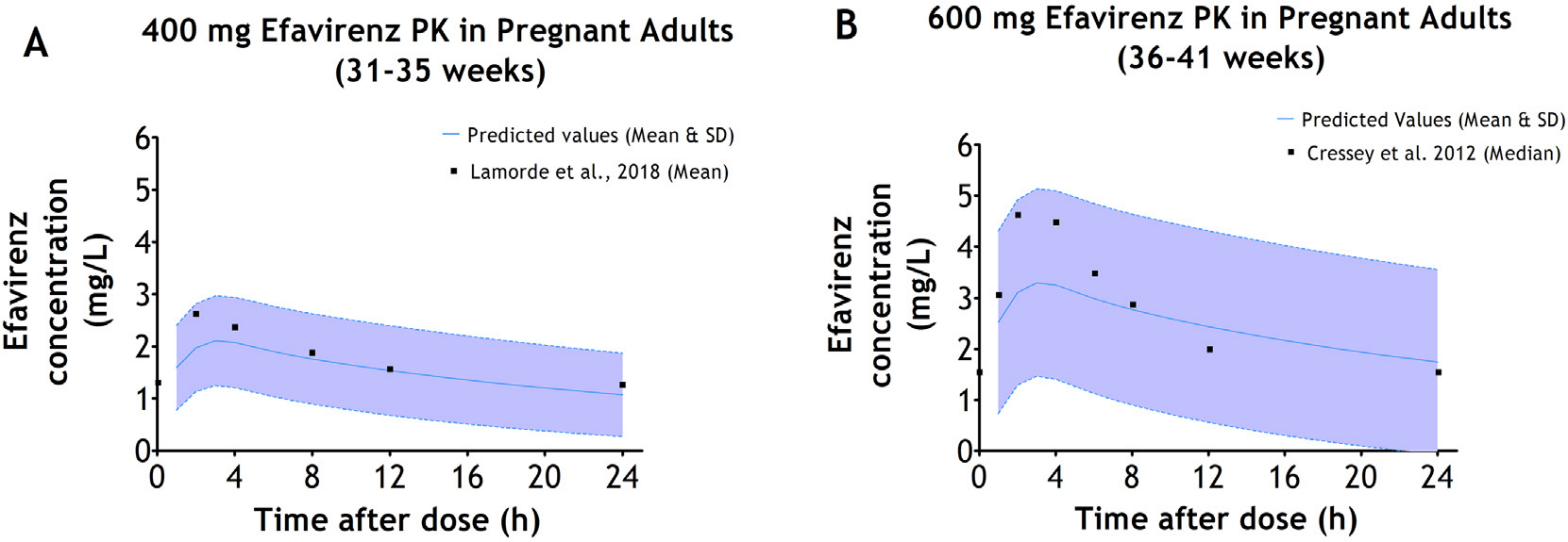
Predicted *vs* Observed plasma concentration-time profile of efavirenz in pregnant adults at third trimester. A - Observed (mean) reported by Lamorde et al. (2018) and Predicted (mean & SD) plasma concentration-time profile of 400 mg efavirenz in pregnant adults at third trimester (31–35 weeks); and B - Observed (median) reported by Cressey et al. (2012) and Predicted (mean & SD) plasma concentration-time profile of 600 mg efavirenz in pregnant adults at third trimester (36–41 weeks).

Conversely, the overlay of the predicted and observed plasma concentration-time profiles of 400 mg and 600 mg efavirenz in pregnant adults at third trimester are shown in Fig. 2. Fig. 2A illustrates the comparison between the mean plasma concentration-time profile of 400 mg efavirenz in pregnant adults during third trimester as reported by Lamorde et al. (2018) and the corresponding predicted plasma concentration-time profile of 400 mg efavirenz in pregnant adults during third trimester. Fig. 2B illustrates the comparison between the median plasma concentration-time profile of 600 mg efavirenz in pregnant adults during third trimester as reported by Cressey et al. (2012) and the corresponding predicted plasma concentration-time profile of 600 mg efavirenz in pregnant adults during third trimester.

### 3.3 Predicted foetal exposure of efavirenz and thalidomide during pregnancy

**Fig. 3:**
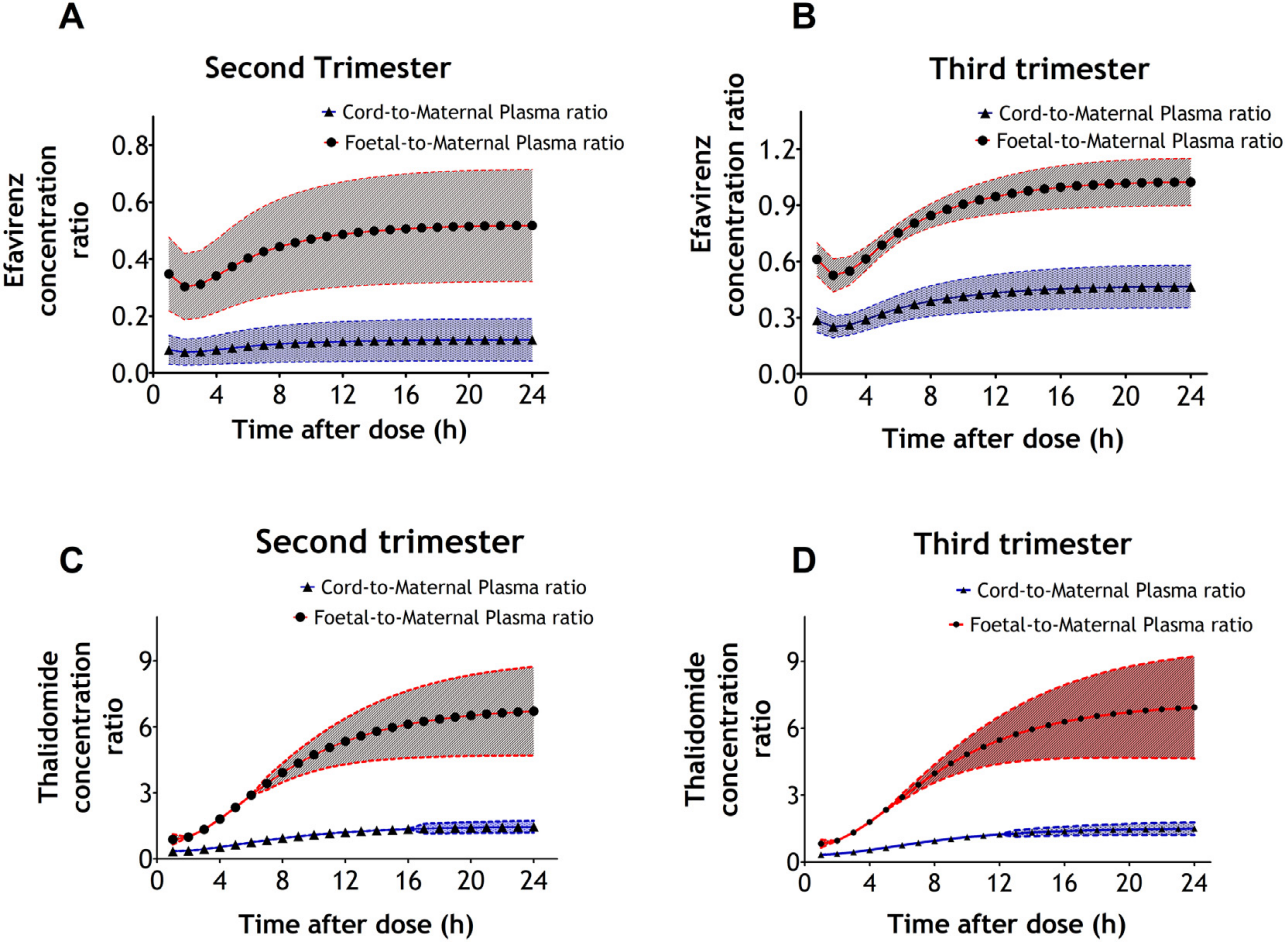
Predicted time profile of cord-to-maternal plasma and foetal-to-maternal plasma concentration ratios of efavirenz and thalidomide across the dosing interval during pregnancy. A - Cord-to-maternal plasma and foetal-to-maternal plasma ratios of efavirenz at second trimester; and B - Cord-to-maternal plasma and foetal-to-maternal plasma ratios of efavirenz at third trimester; C - Cord-to-maternal plasma and foetal-to-maternal plasma ratios of thalidomide at second trimester; and D - Cord-to-maternal plasma and foetal-to-maternal plasma ratios of thalidomide at third trimester. Data presented as mean (SD).

Predicted data on the foetal exposure to efavirenz and thalidomide at second and third trimesters are shown in Table 5. Changes in the foetal-to-maternal and cord-to-maternal plasma ratios (mean and standard deviations) of efavirenz and thalidomide over 24-h dosing interval are presented in Fig. 3. Fig. 3A and B illustrates the predicted ratios for efavirenz at second and third trimesters respectively and Fig. 3C and D illustrates the predicted ratios for thalidomide at second and third trimesters. The figures show that the predicted foetal-to-maternal plasma ratios are consistently higher than the predicted cord-to-maternal plasma ratios. Both predicted cord-to-maternal plasma and foetal-to-maternal plasma ratios are shown to vary across the dosing interval.

**Table 5:** Predicted indices of foetal exposure to efavirenz and thalidomide in the foetal plasma and umbilical cord during pregnancy.

Pharmacokinetic parameter (units)	Second trimester n = 100	Third trimester n = 100	Second trimester n = 100	Third trimester n = 100
Thalidomide	200 mg		400 mg	
Foetal plasma				
Thalidomide concentration (mg/L)	2.15 (1.47–3.56)	2.15 (1.53–3.18)	4.27 (2.68–8.19)	4.31 (3.07–6.37)
AUC_0–24_ (mg.h/L)	51.1 (35.0–84.1)	51.1 (36.5–75.5)	101 (63.8–192)	102 (72.9–151)
F:M	4.69 (3.06–9.57)	4.55 (3.06–9.57)	4.59 (3.21–8.85)	4.55 (3.06–9.57)
Umbilical vein				
Thalidomide concentration (mg/L)	0.537 (0.352–0.899)	0.555 (0.352–0.899)	1.14 (0.729–2.06)	1.11 (0.704–1.80)
AUC_0–24_ (mg.h/L)	12.7 (8.33–21.2)	13.1 (8.33–21.2)	27.0 (17.2–48.5)	26.2 (16.7–42.3)
C:M	1.09 (0.89–1.73)	1.09 (0.89–1.58)	1.05 (0.85–1.54)	1.09 (0.89–1.58)
Efavirenz	400 mg		600 mg	
Foetal plasma				
Efavirenz concentration (mg/L)	0.720 (0.223–2.72)	1.06 (0.499–4.30)	1.02 (0.333–5.65)	1.52 (0.700–6.31)
AUC_0–24_ (mg.h/L)	16.7 (5.19–62.9)	24.7 (11.7–99.2)	23.6 (7.75–130)	35.4 (16.4–145)
F:M	0.47 (0.17–0.74)	0.89 (0.73–1.05)	0.47 (0.17–0.76)	0.89 (0.73–1.06)
Umbilical vein				
Efavirenz concentration (mg/L)	0.159 (0.022–0.869)	0.445 (0.204–1.74)	0.232 (0.033–1.71)	0.642 (0.288–2.56)
AUC_0–24_ (mg.h/L)	3.70 (0.509–20.1)	10.3 (4.75–40.2)	5.36 (0.760–39.3)	14.9 (6.73–59.0)
C:M	0.10 (0.02–0.23)	0.41 (0.25–0.57)	0.10 (0.02–0.23)	0.41 (0.25–0.58)

Data presented as median (range).

## 4 Discussion

A previously validated whole-body oral adult PBPK model was modified to create an adult female PBPK model and a pregnancy PBPK model (Rajoli et al., 2015). The adult female PBPK model was integrated with a multi-compartmental foetal PBPK sub-model and pregnancy-induced changes, mostly defined by gestational age, were also incorporated to give the pregnancy PBPK model. Variability was introduced into the model parameters (drug-specific and system specific parameters) obtained from literature to mimic the variability observed in clinical studies. The fraction of the unbound drug and the level of CYP2B6 enzyme induction by maternal hormones were the only two drug-specific properties that were assumed to be dependent on gestational age. This dependency is due to their association with plasma proteins and estradiol in the plasma respectively during pregnancy (Dallmann et al., 2017; Dickmann and Isoherranen, 2012). Increasing estradiol levels in maternal plasma across gestational age results into increase in estradiol-mediated induction of CYP2B6 between second and third trimesters for the mother but such fluctuation was not considered for the foetus. In addition to the maternal liver having higher CYP2B6 enzyme abundance than the foetal liver, this induction contributes to CYP2B6 enzyme activity being higher in the mother than the foetus for both second and third trimesters. CYP2C19 enzyme activity was higher in the mother than the foetus during pregnancy primarily due to higher abundance of CYP2C19 enzyme in maternal liver compared to foetal liver.

In theory, the foetal-to-maternal plasma ratio may be more suitable for assessing the foetal exposure to maternal drugs as compared to the cord-to-maternal plasma ratio. Foetal-to-maternal plasma ratio relies on the drug concentration in the foetal plasma which may be a better predictor of drug accumulation in the foetus than the cord-to-maternal plasma ratio. Both the cord-to-maternal plasma and foetal-to-maternal plasma ratios were predicted to increase with gestational age when averaged across the dosing interval. The average cord-to-maternal plasma or foetal-to-maternal plasma ratios across each dosing interval would most likely be highest near delivery. This may support the clinical use of cord-to-maternal plasma or foetal-to-maternal plasma ratios at delivery to estimate the highest level of foetal exposure to maternal drugs. However, the validity of this assumption can be influenced by the time difference between the last maternal dose and the time of delivery. Ultimately, the use of cord-to-maternal plasma and foetal-to-maternal plasma ratios obtained at a single time point following the delivery may not sufficiently reflect the highest extent of foetal exposure to maternal drugs as the ratios can vary over the course of the dosing interval. Our simulations indicate that the cord-to-maternal plasma and foetal-to-maternal plasma ratios also vary between different mother-foetus pairs for each given time-point. Interestingly, the predicted cord-to-maternal plasma and foetal-to-maternal plasma ratios are equal across simulated doses as shown for efavirenz and thalidomide in Table 5. This may be explained by the first-order and not saturable nature of the simulated passive diffusion across the placenta utilised for this study. This phenomenon could be particularly useful in scaling the foetal exposure to other doses of the studied drugs.

The average foetal plasma concentration of thalidomide was estimated to be more than 300% that of the maternal concentration compared with 47% and 89% of the maternal plasma concentration for efavirenz during the second and third trimesters, respectively (Table 5). Similarly, the AUC_0–24_ of thalidomide in the foetal plasma was consistently estimated to be higher than the AUC_0–24_ of thalidomide in the maternal plasma, while the AUC_0–24_ of efavirenz in the foetal plasma was estimated to be lower than the maternal plasma during both trimesters. However, the AUC_0–24_ of efavirenz in the foetal plasma was estimated to be higher during the third trimester which could serve to improve the pre-exposure prophylactic effect of efavirenz to the foetus before delivery. The higher foetal exposure for thalidomide could contribute to the higher incidence of teratogenicity but is clearly unlikely to be the sole causative factor. The risk of teratogenicity after *in utero* exposure to thalidomide especially during the time-sensitive window is reported to vary between 50 and 100% (Vargesson, 2015). Meanwhile, an average of 2.9% (0–22.6%) of *in utero* exposure to efavirenz resulted in congenital abnormalities among babies born to women who had been treated with efavirenz during the first trimester which is similar to the incidence of teratogenicity of 2.7% reported for the general population in the US (Correa et al., 2007; Ford et al., 2010). Also, among a compilation of birth defects in 1256 women who were exposed to efavirenz and gave birth to live babies, only one had a neural tube defect (Ford et al., 2010).

There is a current paucity of data on the extent of foetal exposure to most drugs, which has negative implications for evidence-based guidance in determining the risk-benefit for use of drugs in pregnant women. Besides isotretinoin, thalidomide, chloramphenicol, pseudoephedrine, propylthiouracil, glyburide, phenytoin, valproic acid and other drugs well known for their teratogenicity, the number of teratogens will potentially rise with the continuous development and approval of newer drugs. The use of maternofoetal PBPK (mf-PBPK) modelling may help to predict foetal exposure to maternally-administered drugs using virtual pregnant populations as described in this study, but clearly can't help in defining the molecular events that ultimately result in teratogenicity. The model predictions of thalidomide pharmacokinetics in the virtual pregnant population quantitatively shows the foetal exposure to thalidomide when administered to pregnant women and the approach may help quantitate the extent of foetal exposure to other known teratogens, newer drugs and older drugs for which there is a paucity of data. *In silico* or *in vivo* data on the extent of foetal exposure to drugs, especially known teratogens, may help establish a safety threshold that can be employed as a yard-stick to predict the likelihood of foetotoxicity when administered during pregnancy.

The availability of more data defining the anatomical and physiological parameters of the foetus throughout gestation may facilitate the prediction of *in utero* exposure at the first trimester when embryogenesis occurs. The time-sensitive window of damage to thalidomide exposure has been reported as between 20 and 36 days after fertilisation (Vargesson, 2015). Understandably, there were no available clinical data to validate the predicted foetal pharmacokinetics of efavirenz during pregnancy at a time before delivery and the predicted foetal pharmacokinetics of thalidomide both before and at delivery. This model represents a proof-of-concept approach that could be qualified using more drugs considering their corresponding clinical data at delivery. The activity of drug transporters and drug metabolising enzymes in the placenta were not accounted for in the current model. The incorporation of these drug disposition enzymes in the placenta with the expected variation in their activity across gestation would help to further increase the accuracy of model predictions for drugs which are substrates for these enzymes. In addition, the diffusion constants of both efavirenz and thalidomide across the placenta are unknown. The values obtained through sensitivity analysis may not be perfect representations of the actual values. Also, foetal brain was modelled as a single compartment, yielding brain-to-plasma ratio predictions of less than 0.2 for efavirenz and above 3.0 for thalidomide (Table S2). We previously evaluated efavirenz distribution in adult CNS and observed preferential accumulation in brain tissue compared with CSF, at tissue-to-plasma ratios of 15.8 *versus* 0.016, respectively (Curley et al., 2016).

In conclusion, the use of drugs by women of child-bearing age and pregnant women cannot be totally avoided in clinical practice particularly for patients with chronic ailments such as asthma, epilepsy, hypertension, diabetes and HIV. The developed pregnancy PBPK model may play an important role in rationalising the assessment of drug safety through prediction of foetal exposure during pregnancy.

## Funding

This work was supported by a Wellcome Trust Training Fellowship in Public Health and Tropical Medicine to A. Olagunju (204776/Z/16/Z). The funder had no role in study design, data collection and analysis, decision to publish, or preparation of the manuscript.

## Declaration of competing interest

A. Owen and MS have received research grants and/or travel bursaries from Merck, Bristol Myers and Squibb, GlaxoSmithKline, Pfizer, Abbott, ViiV, Boehringer Ingelheim and Janssen Pharmaceuticals. The remaining authors have no competing interests to disclose.

## Figures and Tables

**Fig. S1:**
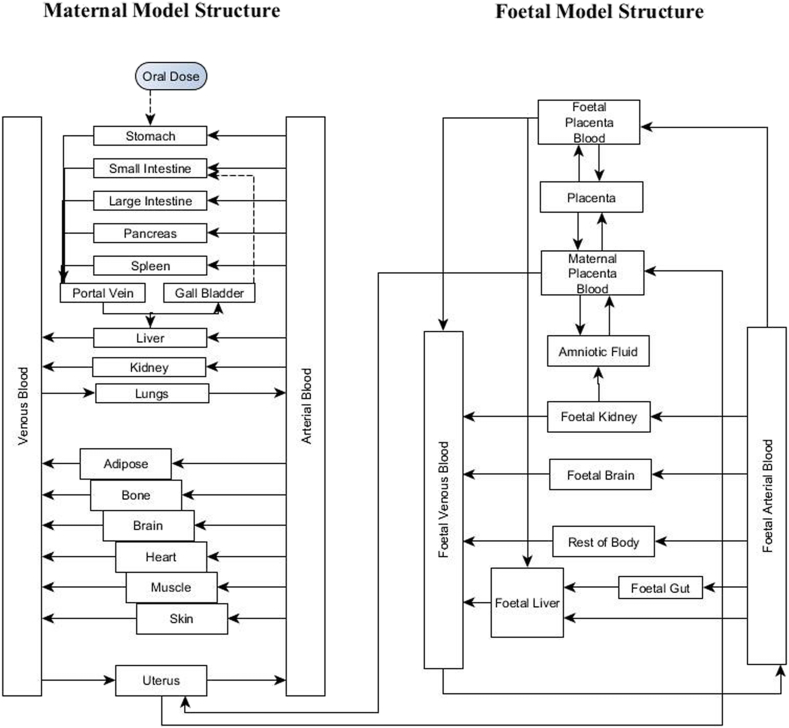
Schematic representation of the mf-PBPK model. Based on a previously described mf-PBPK model structure (Zhang et al., 2017).

## Appendix A Supplementary data

Supplementary tables Image 1

## References

[bb0005] Abduljalil K. (2012). Anatomical, physiological and metabolic changes with gestational age during normal pregnancy: a database for parameters required in physiologically based pharmacokinetic modelling. Clin. Pharmacokinet..

[bb0010] Archie J.G. (2006). Quantitative standards for fetal and neonatal autopsy. Am. J. Clin. Pathol..

[bb0015] Blehar M.C. (2013). Enrolling pregnant women: issues in clinical research. Womens Health Issues.

[bb0020] Bosgra S. (2012). An improved model to predict physiologically based model parameters and their inter-individual variability from anthropometry. Crit. Rev. Toxicol..

[bb0025] Correa A. (2007). Reporting birth defects surveillance data 1968–2003. Birth Defects Res. A Clin. Mol. Teratol..

[bb0030] Cressey T.R. (2012). Efavirenz pharmacokinetics during the third trimester of pregnancy and postpartum. J. Acquir. Immune Defic. Syndr. (1999).

[bb0035] Croom E.L. (2009). Human hepatic CYP2B6 developmental expression: the impact of age and genotype. Biochem. Pharmacol..

[bb0040] Curley P. (2016). Efavirenz is predicted to accumulate in brain tissue: an in silico, in vitro, and in vivo investigation. Antimicrob. Agents Chemother..

[bb0045] Dallmann A. (2017). Physiologically based pharmacokinetic modeling of renally cleared drugs in pregnant women. Clin. Pharmacokinet..

[bb0050] Dickinson L. (2016). Comprehensive pharmacokinetic, pharmacodynamic and pharmacogenetic evaluation of once-daily efavirenz 400 and 600 mg in treatment-naive HIV-infected patients at 96 weeks: results of the ENCORE1 study. Clin. Pharmacokinet..

[bb0055] Dickmann L.J., Isoherranen N. (2012). Quantitative prediction of CYP2B6 induction by estradiol during pregnancy: potential explanation for increased methadone clearance during pregnancy. Drug Metab. Dispos..

[bb0060] FDA (2001). FDA Label: Thalomid Capsules. http://www.accessdata.fda.gov/drugsatfda_docs/label/2001/20785s12s14lbl.pdf.

[bb0065] Ford N. (2010). Safety of efavirenz in first-trimester of pregnancy: a systematic review and meta-analysis of outcomes from observational cohorts. AIDS.

[bb0070] Ford N. (2014). Safety of efavirenz in the first trimester of pregnancy: an updated systematic review and meta-analysis. AIDS.

[bb0075] Gandhi M. (2013). Hair and plasma data show that lopinavir, ritonavir, and efavirenz all transfer from mother to infant in utero, but only efavirenz transfers via breastfeeding. J. Acquir. Immune Defic. Syndr..

[bb0080] Gaohua L. (2012). A pregnancy physiologically based pharmacokinetic (p-PBPK) model for disposition of drugs metabolized by CYP1A2, CYP2D6 and CYP3A4. Br. J. Clin. Pharmacol..

[bb0085] Griffiths S.K., Campbell J.P. (2015). Placental structure, function and drug transfer. Contin. Educ. Anaesth. Crit. Care Pain.

[bb0090] Hines R.N. (2007). Ontogeny of human hepatic cytochromes P450. J. Biochem. Mol. Toxicol..

[bb0095] ICRP (2003). Basic anatomical and physiological data for use in radiological protection. Reference Values.

[bb0100] Karthikeyan T. (2012). Placental thickness & its correlation to gestational age & foetal growth parameters - a cross sectional ultrasonographic study. J. Clin. Diagn. Res..

[bb0105] Lamorde M. (2018). Pharmacokinetics, pharmacodynamics, and pharmacogenetics of efavirenz 400 mg once daily during pregnancy and post-partum. Clin. Infect. Dis..

[bb0110] Lepper E.R. (2006). Thalidomide metabolism and hydrolysis: mechanisms and implications. Curr. Drug Metab..

[bb0115] Lu J. (2004). Metabolism of thalidomide in liver microsomes of mice, rabbits, and humans. J. Pharmacol. Exp. Ther..

[bb0120] Molina D.K., DiMaio V.J. (2015). Normal organ weights in women: part II-the brain, lungs, liver, spleen, and kidneys. Am. J. Forensic Med. Pathol..

[bb0125] Nishiyama S. (2015). Simulation of human plasma concentrations of thalidomide and primary 5-hydroxylated metabolites explored with pharmacokinetic data in humanized tk-nog mice. Chem. Res. Toxicol..

[bb0130] Olagunju A.E. (2015). Pharmacogenetics of Antiretroviral Drugs Used for Prevention of Mother-to-child Transmission of HIV During Pregnancy and Lactation.

[bb0135] Olagunju A. (2015). Pharmacogenetics of pregnancy-induced changes in efavirenz pharmacokinetics. Clin. Pharmacol. Ther..

[bb0140] Olagunju A. (2015). Breast milk pharmacokinetics of efavirenz and breastfed infants' exposure in genetically defined subgroups of mother-infant pairs: an observational study. Clin. Infect. Dis..

[bb0145] Peter S. (2008). Evaluation of a generic physiologically based pharmacokinetic model for lineshape analysis. Clin. Pharmacokinet..

[bb0150] Piscitelli S.C. (1997). Single-dose pharmacokinetics of thalidomide in human immunodeficiency virus-infected patients. Antimicrob. Agents Chemother..

[bb0155] Poulin P., Theil F.P. (2002). Prediction of pharmacokinetics prior to in vivo studies. 1. Mechanism-based prediction of volume of distribution. J. Pharm. Sci..

[bb0160] Rajoli R.K. (2015). Physiologically based pharmacokinetic modelling to inform development of intramuscular long-acting nanoformulations for HIV. Clin. Pharmacokinet..

[bb0165] Siccardi M. (2013). Use of a physiologically-based pharmacokinetic model to simulate artemether dose adjustment for overcoming the drug-drug interaction with efavirenz. In Silico Pharmacol..

[bb0170] Sutton M.S. (1994). Assessment of changes in blood flow through the lungs and foramen ovale in the normal human fetus with gestational age: a prospective Doppler echocardiographic study. Br. Heart J..

[bb0175] Vargesson N. (2009). Thalidomide-induced limb defects: resolving a 50-year-old puzzle. BioEssays.

[bb0180] Vargesson N. (2015). Thalidomide-induced teratogenesis: history and mechanisms. Birth Defects Res. C.

[bb0185] Villani P. (1999). Pharmacokinetics of efavirenz (EFV) alone and in combination therapy with nelfinavir (NFV) in HIV-1 infected patients. Br. J. Clin. Pharmacol..

[bb0190] WHO (2009). A systematic review of the teratogenicity of efavirenz. WHO ART Guidelines Meeting Review, October 2009.

[bb0195] WHO (2016). Consolidated Guidelines on the Use of Antiretroviral Drugs for Treating and Preventing HIV Infection: Recommendations for a Public Health Approach.

[bb0200] Yu L.X., Amidon G.L. (1999). A compartmental absorption and transit model for estimating oral drug absorption. Int. J. Pharm..

[bb0205] Zhang Z., Imperial M.Z., Patilea-Vrana G.I., Wedagedera J., Gaohua L., Unadkat J.D. (2017). Development of a novel maternal-fetal physiologically based pharmacokinetic model I: insights into factors that determine fetal drug exposure through simulations and sensitivity analyses. Drug Metab. Dispos..

