# Supplementary material for: Using mechanistic physiologically-based pharmacokinetic models to assess prenatal drug exposure: Thalidomide versus efavirenz as case studies
Source: Eur J Pharm Sci. 2019 Dec 1;140:105068. doi: 10.1016/j.ejps.2019.105068 (PMC6853277; doi:10.1016/j.ejps.2019.105068)
Supplement: Supplementary file 1 — Supplementary tables [file mmc1.docx]

**SUPPLEMENTARY MATERIALS**

**Table S1.** Sensitivity analysis of efavirenz passive diffusion constant (K, x 10 cm^2^/s)

| PK parameters | | **Diffusion constant (K) n = 50** | | | | | | | | | | | | | |
| --- | --- | --- | --- | --- | --- | --- | --- | --- | --- | --- | --- | --- | --- | --- | --- |
| Observed Umbilical Vein EFV conc Summary | | 0.125 | | 0.25 | | 0.5 | | 1 | | 2 | | 4 | | 10 | |
|  |  | Pred | %PE | Pred | %PE | Pred | %PE | Pred | %PE | Pred | PE | Pred | %PE | Pred | %PE |
|  | mg/L | mg/L | % | mg/L | % | mg/L | % | mg/L | % | mg/L | % | mg/L | % | mg/L | % |
| Median | 1.05 | 0.531 | -46.93 | 0.684 | -31.61 | 0.934 | -6.59 | 1.134 | 13.35 | 1.225 | 22.48 | 1.485 | 48.48 | 1.309 | 30.93 |
| Lower | 0.47 | 0.294 | -70.63 | 0.260 | -73.99 | 0.375 | -62.46 | 0.520 | -47.99 | 0.421 | -57.87 | 0.660 | -34.00 | 0.489 | -51.09 |
| Upper | 4.51 | 3.225 | 222.48 | 4.240 | 324.01 | 5.644 | 464.43 | 7.429 | 642.89 | 6.669 | 566.85 | 6.840 | 583.99 | 6.699 | 569.85 |
| Observed Foetal Plasma EFV conc Summary | | 0.125 | | 0.25 | | 0.5 | | 1 | | 2 | | 4 | | 10 | |
|  |  | Pred | %PE | Pred | %PE | Pred | %PE | Pred | %PE | Pred | PE | Pred | %PE | Pred | %PE |
|  | mg/L | mg/L | % | mg/L | % | mg/L | % | mg/L | % | mg/L | % | mg/L | % | mg/L | % |
| Median | 1.695 | 1.036 | 3.56 | 1.332 | 33.17 | 1.836 | 83.62 | 2.191 | 119.10 | 2.331 | 133.06 | 2.892 | 189.16 | 2.583 | 158.32 |
| Lower | 0.050 | 0.593 | -40.73 | 0.535 | -46.50 | 0.722 | -27.77 | 0.995 | -0.47 | 0.823 | -17.70 | 1.255 | 25.53 | 0.946 | -5.44 |
| Upper | 7.881 | 6.477 | 547.69 | 8.434 | 743.40 | 11.303 | 1030.28 | 14.862 | 1386.22 | 12.647 | 1164.65 | 12.941 | 1194.15 | 13.246 | 1224.62 |
|  |  |  | |  | |  | |  | |  | |  | |  | |
| Prediction precision | | 932.01 | | 1252.68 | | 1675.14 | | 2210.02 | | 1962.61 | | 2075.31 | | 2040.25 | |
| Prediction bias | | 615.44 | | 948.48 | | 1481.51 | | 2113.10 | | 1811.48 | | 2007.31 | | 1927.19 | |
| Sum of Squares | | 4.65 | | 0.92 | | 13.49 | | 58.41 | | 28.40 | | 34.15 | | 35.23 | |

**Table S2.** Predicted indices of foetal exposure to efavirenz and thalidomide in the foetal brain during pregnancy.

| **Pharmacokinetic Parameter (Units)** | **Second Trimester**  **n = 100** | **Third Trimester**  **n = 100** | **Second Trimester**  **n = 100** | **Third Trimester**  **n = 100** |
| --- | --- | --- | --- | --- |
| **Thalidomide** | **200 mg** | | **400 mg** | |
| **Foetal Brain** |  |  |  |  |
| Thalidomide concentration (mg/L) | 2.15 (1.48-3.56) | 2.16 (1.54-3.19) | 4.28 (2.68-8.18) | 4.31 (3.07-6.37) |
| AUC_0-24_ (mg.h/L) | 51.1 (35.1-84.0) | 51.1 (36.5-75.6) | 101 (63.8-193) | 102 (73.0-151) |
| Foetal brain-to-maternal plasma ratio | 4.71 (3.07-9.60) | 4.56 (3.07-9.60) | 4.61 (3.22-8.88) | 4.56 (3.07-9.60) |
| **Efavirenz** | **400 mg** | | **600 mg** | |
| **Foetal Brain** |  |  |  |  |
| Efavirenz concentration (mg/L) | 0.073 (0.023-0.275) | 0.107 (0.05-0.433) | 0.104 (0.035-0.571) | 0.153 (0.07-0.635) |
| AUC_0-24_ (mg.h/L) | 1.69 (0.542-6.35) | 2.49 (1.17-9.99) | 2.41 (0.809-13.2) | 3.57 (1.64-14.7) |
| Foetal brain-to-maternal plasma ratio | 0.05 (0.02-0.07) | 0.09 (0.07-1.10) | 0.05 (0.02-0.08) | 0.09 (0.07-0.11) |

Data presented as median (range)
